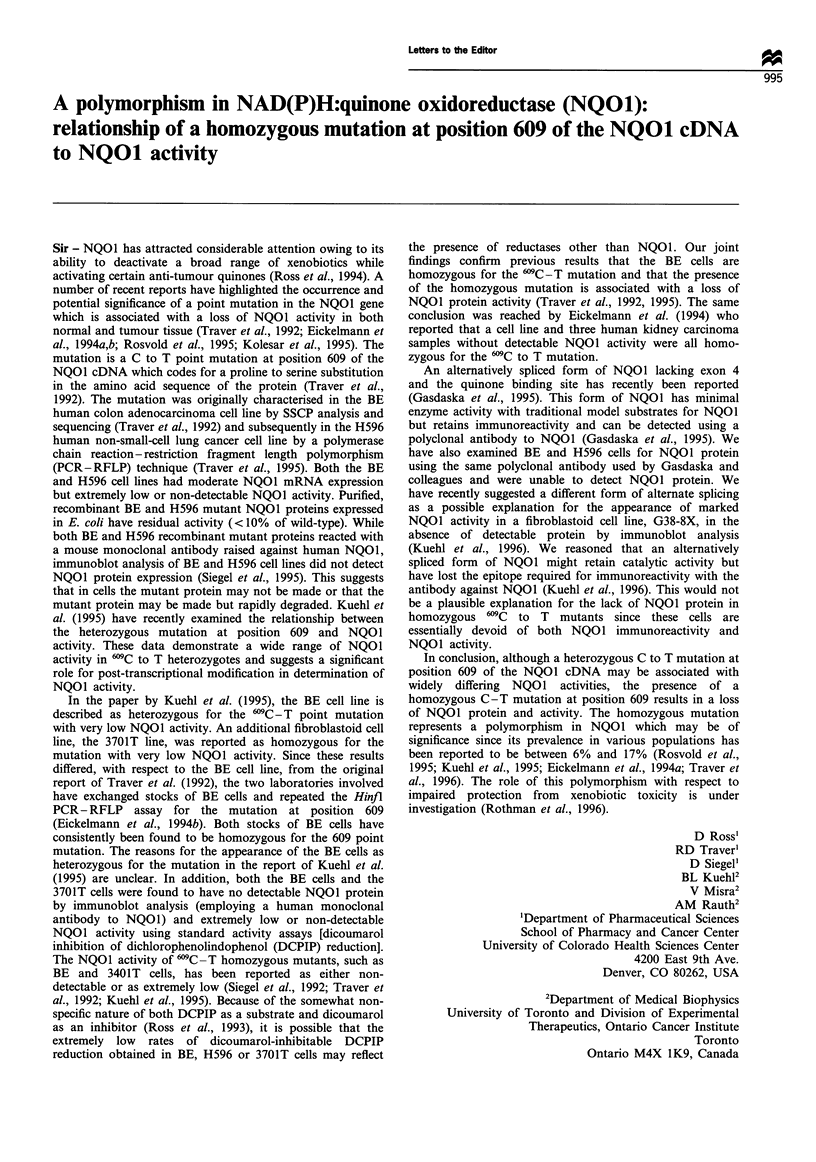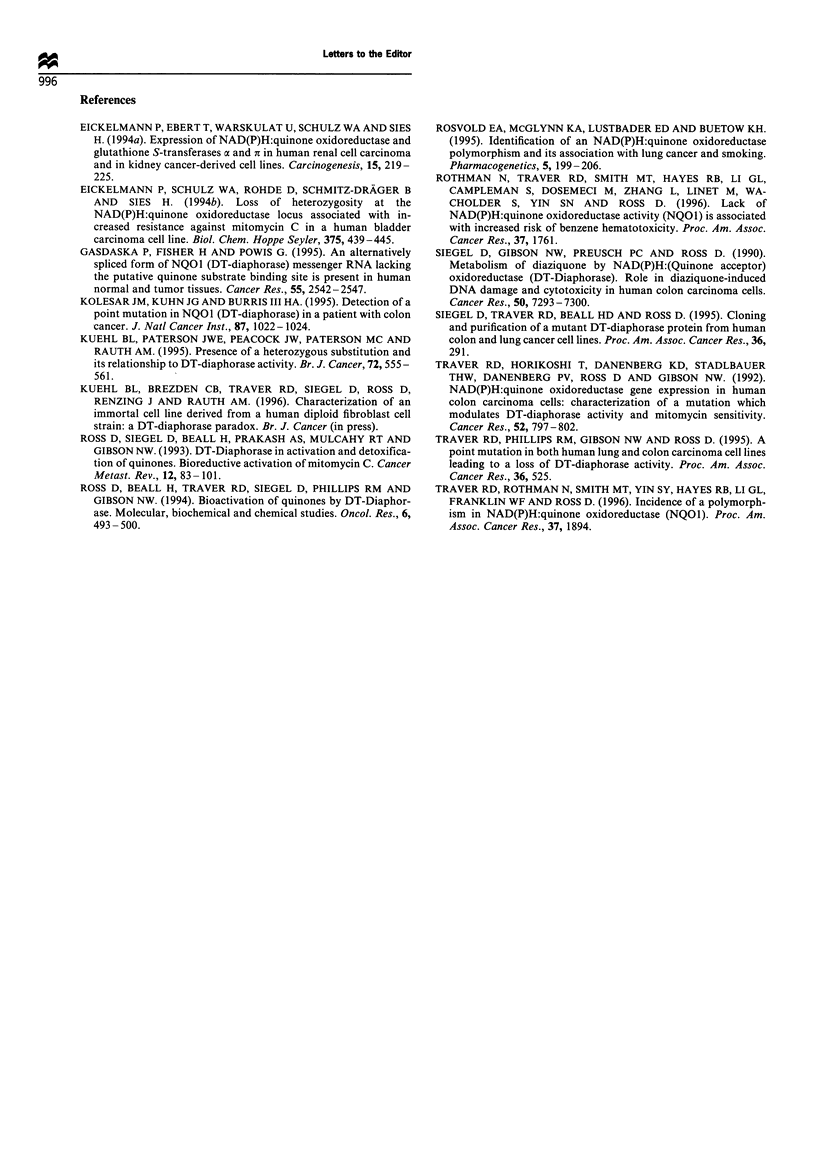# A polymorphism in NAD(P)H:quinone oxidoreductase (NQO1): relationship of a homozygous mutation at position 609 of the NQO1 cDNA to NQO1 activity.

**DOI:** 10.1038/bjc.1996.477

**Published:** 1996-09

**Authors:** D. Ross, R. D. Traver, D. Siegel, B. L. Kuehl, V. Misra, A. M. Rauth


### A polymorphism in NAD(P)H:quinone oxidoreductase (NQO1): relationship of a homozygous mutation at position 609 of the NQO1 cDNA to NQO1 activity

**Sir** – NQO1 has attracted considerable attention owing to its ability to deactivate a broad range of xenobiotics while activating certain anti-tumour quinones (Ross *et al.*, 1994). A number of recent reports have highlighted the occurrence and potential significance of a point mutation in the NQO1 gene which is associated with a loss of NQO1 activity in both normal and tumour tissue (Traver *et al.*, 1992; Eickelmann *et al.*, 1994*a*,*b*; Rosvold *et al.*, 1995; Kolesar *et al.*, 1995). The mutation is a C to T point mutation at position 609 of the NQO1 cDNA which codes for a proline to serine substitution in the amino acid sequence of the protein (Traver *et al.*, 1992). The mutation was originally characterised in the BE human colon adenocarcinoma cell line by SSCP analysis and sequencing (Traver *et al.*, 1992) and subsequently in the H596 human non-small-cell lung cancer cell line by a polymerase chain reaction–restriction fragment length polymorphism (PCR–RFLP) technique (Traver *et al.*, 1995). Both the BE and H596 cell lines had moderate NQO1 mRNA expression but extremely low or non-detectable NQO1 activity. Purified, recombinant BE and H596 mutant NQO1 proteins expressed in *E. coli* have residual activity (<10% of wild-type). While both BE and H596 recombinant mutant proteins reacted with a mouse monoclonal antibody raised against human NQO1, immunoblot analysis of BE and H596 cell lines did not detect NQO1 protein expression (Siegel *et al.*, 1995). This suggests that in cells the mutant protein may not be made or that the mutant protein may be made but rapidly degraded. Kuehl *et al.* (1995) have recently examined the relationship between the heterozygous mutation at position 609 and NQO1 activity. These data demonstrate a wide range of NQO1 activity in $^{609}$C to T heterozygotes and suggests a significant role for post-transcriptional modification in determination of NQO1 activity.

In the paper by Kuehl *et al.* (1995), the BE cell line is described as heterozygous for the $^{609}$C–T point mutation with very low NQO1 activity. An additional fibroblastoid cell line, the 3701T line, was reported as homozygous for the mutation with very low NQO1 activity. Since these results differed, with respect to the BE cell line, from the original report of Traver *et al.* (1992), the two laboratories involved have exchanged stocks of BE cells and repeated the *Hinf*I PCR–RFLP assay for the mutation at position 609 (Eickelmann *et al.*, 1994*b*). Both stocks of BE cells have consistently been found to be homozygous for the 609 point mutation. The reasons for the appearance of the BE cells as heterozygous for the mutation in the report of Kuehl *et al.* (1995) are unclear. In addition, both the BE cells and the 3701T cells were found to have no detectable NQO1 protein by immunoblot analysis (employing a human monoclonal antibody to NQO1) and extremely low or non-detectable NQO1 activity using standard activity assays [dicoumarol inhibition of dichlorophenolindophenol (DCPIP) reduction]. The NQO1 activity of $^{609}$C–T homozygous mutants, such as BE and 3401T cells, has been reported as either non-detectable or as extremely low (Siegel *et al.*, 1992; Traver *et al.*, 1992; Kuehl *et al.*, 1995). Because of the somewhat non-specific nature of both DCPIP as a substrate and dicoumarol as an inhibitor (Ross *et al.*, 1993), it is possible that the extremely low rates of dicoumarol-inhibitable DCPIP reduction obtained in BE, H596 or 3701T cells may reflect the presence of reductases other than NQO1. Our joint findings confirm previous results that the BE cells are homozygous for the $^{609}$C–T mutation and that the presence of the homozygous mutation is associated with a loss of NQO1 protein activity (Traver *et al.*, 1992, 1995). The same conclusion was reached by Eickelmann *et al.* (1994) who reported that a cell line and three human kidney carcinoma samples without detectable NQO1 activity were all homozygous for the $^{609}$C to T mutation.

An alternatively spliced form of NQO1 lacking exon 4 and the quinone binding site has recently been reported (Gasdaska *et al.*, 1995). This form of NQO1 has minimal enzyme activity with traditional model substrates for NQO1 but retains immunoreactivity and can be detected using a polyclonal antibody to NQO1 (Gasdaska *et al.*, 1995). We have also examined BE and H596 cells for NQO1 protein using the same polyclonal antibody used by Gasdaska and colleagues and were unable to detect NQO1 protein. We have recently suggested a different form of alternate splicing as a possible explanation for the appearance of marked NQO1 activity in a fibroblastoid cell line, G38-8X, in the absence of detectable protein by immunoblot analysis (Kuehl *et al.*, 1996). We reasoned that an alternatively spliced form of NQO1 might retain catalytic activity but have lost the epitope required for immunoreactivity with the antibody against NQO1 (Kuehl *et al.*, 1996). This would not be a plausible explanation for the lack of NQO1 protein in homozygous $^{609}$C to T mutants since these cells are essentially devoid of both NQO1 immunoreactivity and NQO1 activity.

In conclusion, although a heterozygous C to T mutation at position 609 of the NQO1 cDNA may be associated with widely differing NQO1 activities, the presence of a homozygous C–T mutation at position 609 results in a loss of NQO1 protein and activity. The homozygous mutation represents a polymorphism in NQO1 which may be of significance since its prevalence in various populations has been reported to be between 6% and 17% (Rosvold *et al.*, 1995; Kuehl *et al.*, 1995; Eickelmann *et al.*, 1994*a*; Traver *et al.*, 1996). The role of this polymorphism with respect to impaired protection from xenobiotic toxicity is under investigation (Rothman *et al.*, 1996).

D Ross[1]
RD Traver[1]
D Siegel[1]
BL Kuehl[2]
V Misra[2]
AM Rauth[2]
[1]Department of Pharmaceutical Sciences
School of Pharmacy and Cancer Center
University of Colorado Health Sciences Center
4200 East 9th Ave.
Denver, CO 80262, USA

[2]Department of Medical Biophysics
University of Toronto and Division of Experimental
Therapeutics, Ontario Cancer Institute
Toronto
Ontario M4X 1K9, Canada

### References

EICKELMANN P, EBERT T, WARSKULAT U, SCHULZ WA AND SIES H. (1994*a*). Expression of NAD(P)H:quinone oxidoreductase and glutathione *S*-transferases $\alpha$ and $\pi$ in human renal cell carcinoma and in kidney cancer-derived cell lines. *Carcinogenesis*, **15**, 219–225.

EICKELMANN P, SCHULZ WA, ROHDE D, SCHMITZ-DRÄGER B AND SIES H. (1994*b*). Loss of heterozygosity at the NAD(P)H:quinone oxidoreductase locus associated with increased resistance against mitomycin C in a human bladder carcinoma cell line. *Biol. Chem. Hoppe Seyler*, **375**, 439–445.

GASDASKA P, FISHER H AND POWIS G. (1995). An alternatively spliced form of NQO1 (DT-diaphorase) messenger RNA lacking the putative quinone substrate binding site is present in human normal and tumor tissues. *Cancer Res.*, **55**, 2542–2547.

KOLESAR JM, KUHN JG AND BURRIS III HA. (1995). Detection of a point mutation in NQO1 (DT-diaphorase) in a patient with colon cancer. *J. Natl Cancer Inst.*, **87**, 1022–1024.

KUEHL BL, PATERSON JWE, PEACOCK JW, PATERSON MC AND RAUTH AM. (1995). Presence of a heterozygous substitution and its relationship to DT-diaphorase activity. *Br. J. Cancer*, **72**, 555–561.

KUEHL BL, BREZDEN CB, TRAVER RD, SIEGEL D, ROSS D, RENZING J AND RAUTH AM. (1996). Characterization of an immortal cell line derived from a human diploid fibroblast cell strain: a DT-diaphorase paradox. *Br. J. Cancer* (in press).

ROSS D, SIEGEL D, BEALL H, PRAKASH AS, MULCAHY RT AND GIBSON NW. (1993). DT-Diaphorase in activation and detoxification of quinones. Bioreductive activation of mitomycin C. *Cancer Metast. Rev.*, **12**, 83–101.

ROSS D, BEALL H, TRAVER RD, SIEGEL D, PHILLIPS RM AND GIBSON NW. (1994). Bioactivation of quinones by DT-Diaphorase. Molecular, biochemical and chemical studies. *Oncol. Res.*, **6**, 493–500.

ROSVOLD EA, MCGLYNN KA, LUSTBADER ED AND BUETOW KH. (1995). Identification of an NAD(P)H:quinone oxidoreductase polymorphism and its association with lung cancer and smoking. *Pharmacogenetics*, **5**, 199–206.

ROTHMAN N, TRAVER RD, SMITH MT, HAYES RB, LI GL, CAMPLEMAN S, DOSEMECI M, ZHANG L, LINET M, WACHOLDER S, YIN SN AND ROSS D. (1996). Lack of NAD(P)H:quinone oxidoreductase activity (NQO1) is associated with increased risk of benzene hematotoxicity. *Proc. Am. Assoc. Cancer Res.*, **37**, 1761.

SIEGEL D, GIBSON NW, PREUSCH PC AND ROSS D. (1990). Metabolism of diaziquone by NAD(P)H:(Quinone acceptor) oxidoreductase (DT-Diaphorase). Role in diaziquone-induced DNA damage and cytotoxicity in human colon carcinoma cells. *Cancer Res.*, **50**, 7293–7300.

SIEGEL D, TRAVER RD, BEALL HD AND ROSS D. (1995). Cloning and purification of a mutant DT-diaphorase protein from human colon and lung cancer cell lines. *Proc. Am. Assoc. Cancer Res.*, **36**, 291.

TRAVER RD, HORIKOSHI T, DANENBERG KD, STADLBAUER THW, DANENBERG PV, ROSS D AND GIBSON NW. (1992). NAD(P)H:quinone oxidoreductase gene expression in human colon carcinoma cells: characterization of a mutation which modulates DT-diaphorase activity and mitomycin sensitivity. *Cancer Res.*, **52**, 797–802.

TRAVER RD, PHILLIPS RM, GIBSON NW AND ROSS D. (1995). A point mutation in both human lung and colon carcinoma cell lines leading to a loss of DT-diaphorase activity. *Proc. Am. Assoc. Cancer Res.*, **36**, 525.

TRAVER RD, ROTHMAN N, SMITH MT, YIN SY, HAYES RB, LI GL, FRANKLIN WF AND ROSS D. (1996). Incidence of a polymorphism in NAD(P)H:quinone oxidoreductase (NQO1). *Proc. Am. Assoc. Cancer Res.*, **37**, 1894.